# Glycogen Synthase Kinase-3β Inhibition Links Mitochondrial Dysfunction, Extracellular Matrix Remodelling and Terminal Differentiation in Chondrocytes

**DOI:** 10.1038/s41598-017-12129-5

**Published:** 2017-09-21

**Authors:** S. Guidotti, M. Minguzzi, D. Platano, S. Santi, G. Trisolino, G. Filardo, E. Mariani, R. M. Borzì

**Affiliations:** 1 Laboratorio di Immunoreumatologia e Rigenerazione Tessutale, Istituto Ortopedico Rizzoli, Bologna, Italy; 2 Dipartimento di Scienze Mediche e Chirurgiche, Università di Bologna, Bologna, Italy; 3 Istituto di Genetica Molecolare, CNR - Laboratorio di Biologia Cellulare Muscoloscheletrica, Istituto Ortopedico Rizzoli, Bologna, Italy; 4 Chirurgia ricostruttiva articolare dell’anca e del ginocchio, Istituto Ortopedico Rizzoli, Bologna, Italy; 5 Clinica Ortopedica e Traumatologica II/Laboratorio di NanoBiotecnologie, Istituto Ortopedico Rizzoli, Bologna, Italy

## Abstract

Following inflammatory stimuli, GSK3 inhibition functions as a hub with pleiotropic effects leading to cartilage degradation. However, little is known about the effects triggered by its direct inhibition as well as the effects on mitochondrial pathology, that contributes to osteoarthritis pathogenesis. To this aim we assessed the molecular mechanisms triggered by GSK3β inactivating stimuli on 3-D (micromass) cultures of human articular chondrocytes. Stimuli were delivered either at micromass seeding (long term) or after maturation (short term) to explore “late” effects on terminal differentiation or “early” mitochondrial effects, respectively. GSK3β inhibition significantly enhanced mitochondrial oxidative stress and damage and endochondral ossification based on increased nuclear translocation of Runx-2 and β-catenin, calcium deposition, cell death and enhanced remodelling of the extracellular matrix as demonstrated by the increased collagenolytic activity of supernatants, despite unmodified (MMP-1) or even reduced (MMP-13) collagenase gene/protein expression. Molecular dissection of the underlying mechanisms showed that GSK3β inhibition achieved with pharmacological/silencing strategies impacted on the control of collagenolytic activity, via both decreased inhibition (reduced TIMP-3) and increased activation (increased MMP-10 and MMP-14). To conclude, the inhibition of GSK3β enhances terminal differentiation via concerted effects on ECM and therefore its activity represents a tool to keep articular cartilage homeostasis.

## Introduction

Healthy articular chondrocytes are post-mitotic cells expected to survive for several years in a maturation arrested state which only requires a low homeostatic turnover of extracellular matrix (ECM) proteins. An intact ECM delivers survival signal to chondrocytes^1^ while, conversely, proteolytic enzymes leads to production of bioactive molecules that promote chondrocyte differentiation, thus boosting osteoarthritis (OA) pathogenesis^2,3^.

GSK3 is among the molecular constraints that keep chondrocytes in a “maturational arrested state”^4^ preventing β-catenin activation (dephosphorylation), its nuclear translocation and subsequent transcriptional activation of TCF/LEF complex.

The relevance of this mechanism in OA development has been pointed out by conditional activation of β-catenin in mouse articular chondrocytes, that led to cartilage destruction and accelerated progression towards terminal differentiation^5^. On the other hand, conditional complete ablation of β-catenin signaling pathway has been associated with cartilage degeneration in transgenic mice^6^, but due to a significant increase in articular chondrocyte apoptosis. Therefore, healthy articular cartilage requires an “housekeeping“ level of β-catenin signaling maintained via fine tuning of GSK3^4^.

There are two GSK3 isoforms, α and β, that despite some redundancy exert tissue^7^ and signaling^8^ specific roles in the cells. Although both isoforms contribute to skeleton formation, GSK3β is the only GSK3 protein expressed in articular chondrocytes in healthy cartilage^9^. Moreover, findings of functional genomics studies on global knockout mice indicate that GSK3β^10^ plays a more important role in skeletal development compared to GSK3α^11^.

Inhibition of GSK3α/β via serine 21/9 phosphorylation and subsequent β-catenin activation is a key event in chondrocyte differentiation in the context of endochondral ossification, a process that is recapitulated in OA. Indeed, Miclea and coworkers showed that, in rats, intra-articular injection of a selective GSK3 inhibitor induces OA changes in articular cartilage^12^. In endochondral ossification, a number of different regulatory kinases affect GSK3β phosphorylation status and drive the process towards hypertrophy and terminal differentiation. Akt has been reported to regulate skeletal development through GSK3, mTOR and FoxOs^13^. In growth plate undergoing endochondral ossification, the inactivating GSK3β phosphorylation is instead due to cGMP dependent protein kinase II responsible for chondrocyte hypertrophic differentiation^14^.

Recently, Litherland and co-workers have established that GSK3 inhibition, at the cross of several inflammatory networks, occurs following different inflammatory stimuli and is responsible for enhanced cartilage destruction in a murine DMM model^15^. This enhanced ECM catabolism is likely due to increased activation/decreased inhibition of matrix degrading enzymes, despite differential effects on MMP gene and protein expression. In this context, the effects of inflammatory cytokines on ECM catabolism were worsened by the delivery of GSK3 inhibitors which conversely had been previously proposed as a potential therapeutic tool in OA^16–18^ because of their anti-NF-κB or p38 inhibiting activity^18^. Moreover, recent findings have pointed at a role of inhibition of “mitochondrial GSK3” in reactive oxygen species (ROS) generation, DNA damage and cell senescence in exponentially growing cells^19,20^. In this setting we had recently shown that GSK3 inhibition links oxidative damage, hypertrophy and senescence, mimicking the status of chondrocytes in cartilage of obese OA patients^20^.

Since GSK3β inhibition has been linked to ECM remodelling we aimed at analyzing its effects on several aspects of terminal differentiation using primary knee OA chondrocytes cultured in 3-D (micromasses) in order to improve the biological relevance of the findings^21^. Grown in micromasses, chondrocytes recover a healthy articular phenotype in few days and become surrounded by their native ECM^22^. Moreover, 3-D culture appears as a convenient surrogate for chondrocyte “maturation” that reproduces dynamically^23^ and is therefore suitable to evaluate the effects on cells and matrix protein of key signalling intermediates or culture conditions^24–27^. At first, we checked the differential expression of the α and the β isoforms in human articular chondrocytes grown in 3-D culture, since previous studies carried out with exponentially growing monolayer indicated the presence of both isoforms, almost equally expressed^15^. We then analyzed the effects of different GSK3β inhibitors on mitochondrial health (potential, production of ROS and oxidative damage to mitochondrial DNA), nuclear translocation of transcription factors pivotal for endochondral ossification, cell viability and global reprogramming of matrix degrading enzymes at transcriptional, translational and post translational level. Particularly we focused on the control of activation/inhibition of matrix degrading enzymes that ultimately drives endochondral ossification and its recapitulation in OA.

We conclude that GSK3β activity is a requirement for a healthy articular cartilage since it impacts on chondrocyte viability, mitochondrial function and ECM integrity and therefore is a fundamental factor for “maturational arrest” maintenance.

## Materials and Methods

All methods and experiments were performed in accordance with the relevant guidelines and regulations.

### GSK3 inhibition

GSK3 inhibition was achieved with 5 mM LiCl or 10 µM SB216763 or 33 nM insulin. Moreover, confirmation of the specificity of the effects was also obtained with small interfering RNA-mediated gene silencing of GSK3β, as in^20^.

### Chondrocyte cultures

After approval of the Ethics Committee of Rizzoli Orthopaedic Institute and patients’ informed consent, primary chondrocytes isolated with sequential enzymatic digestion were obtained from 20 OA patients undergoing knee arthroplasty, and seeded in 3-D (micromass) culture essentially as described in^24^. The GSK3β inactivating stimuli were either delivered “long term” (at micromass seeding and medium changes during its maturation, up to 1week) to test the effects on chondrocyte hypertrophy and terminal differentiation or “short term” (for 8, 16 and 24 hours) after micromass maturation to evaluate early effects. Micromasses were then used for immunohistochemistry or immunofluorescence, western blot or real time PCR or mineralization assays essentially as described in^24^. Some samples were instead employed for live sample imaging as described below. Supernatants were collected either in regular medium or after an overnight starvation in order to avoid serum interference on electrophoresis and/or enzymatic activity assessment.

### Assessment of the differential expression and phosphorylation of the two GSK3 isoforms

The expression pattern of the two GSK3 isoforms was investigated by means of western blot analysis using an anti-GSK3α/β antibody (the ProteinAtlas validated antibody sc-7291, Santa Cruz Biotechnology). Protein lysates corresponding to half micromass or 150000 cells of exponentially growing monolayer were loaded and processed as described in^28^. Signals were revealed with ECL Select kit (GE Healthcare) as in^20^. Moreover, we tested the effect of the GSK3β inactivating stimuli on both isoforms using the anti-﻿phosphoGSK3α/β antibody (Cell Signaling #9331). In some experiments GSK3β phosphorylation was also assessed by mean of an anti-phosphoGSK3β rabbit monoclonal antibody (Cell Signaling #5558) used as in^20^. Anti-GAPDH (clone 6C5, Chemicon–Millipore) served as loading control.

### Evaluation of the mitochondrial effects of “short term” GSK3β inhibition

A first investigation was performed using MitoTracker dye (MitoTracker Orange CMTMRos, Invitrogen), applied at 200 nM in serum-free medium (30 min at 37 °C) to viable micromasses established with cells of two different patients and treated with GSK3β inhibiting stimuli. The staining intensity of this probe has been shown to sensibly and reliably evidence changes in transmembrane potential as well as increased generation of ROS^29^. Change in MitoTracker staining was investigated exploiting the use of Light Sheet technology (Zeiss) that allows for “live” sample imaging of almost the entire samples^30^. Hoechst 33342 was used as general counterstaining, while nuclei of dead cells, unable to exclude the dye, were stained by mean of Sytox Green.

ROS generation was assessed by using MitoSox probe for superoxide (Invitrogen), essentially as indicated by the manufacturer and exploiting flow cytometric detection with a FACSCanto II (Becton Dickinson). Live cells were obtained by mild enzymatic digestion of micromasses treated with GSK3β inactivating stimuli for 16 hours. Each condition was carried out in triplicate and the experiments were run with cells from three different patients. Half of the cells derived from each micromass were used as a background fluorescence control and subtracted from MitoSox fluorescence to obtain the Mean channel of Fluorescence intensity Increment (MCFI).

Overlapped signals of MitoTracker Orange CMTMRos and LysoTracker Green (Invitrogen) were evaluated by Light Sheet technology in live micromass samples to assess occurrence of mitophagy^31^.

A confirmation of ROS generation leading to cell damage and death was obtained by staining the samples with 30 µM 2′,7′-Dichlorofluorescein diacetate (DCHF-DA), a robust indicator of generalized oxidative stress^32^, in conjunction with the dead cell staining ethidium homodimer.

MitoTracker was also applied to micromass sections, exploiting its ability to bind heat shock proteins that increase after mitochondrial activation^33^. The extent of mitochondrial oxidative damage at 16 hours stimulation was investigated by mean of staining of 8-oxo-dG (Trevigen), a well known oxidative damage marker^34^, performed by immunohistochemistry and image analysis of the samples, essentially as described in^35^. Mitochondrial localization of 8-oxo-dG was confirmed by combining the immunofluorescent staining of 8-oxo-dG (detected by an Anti-Mouse Alexa Fluor 555) with that of the mitochondrial marker Tom20 (Santa Cruz Biotechnology, detected by Streptavidin Alexa Fluor 488), thus yielding an orange signal. A 3-D reconstruction of one representative cell for each condition was undertaken and a quantitative colocalization analysis carried out using the overlap coefficient K2, in order to compare the different culture conditions.

The consequences of mitochondrial oxidative stress following GSK3β inhibition achieved by either pharmacologic or silencing strategies were investigated by mean of the luminescent assessment of increased activity of major initiator (caspase 8 and 9) and effector (3 and 6) caspases by using caspase-Glo reagents (Promega). To evaluate the extent of caspase activation, micromasses were lysed with 20 µl of RIPA buffer^20^. 2 µl of the extract were diluted with PBS to the volume of 75 µl, joined to the same volume of substrate and left to incubate for 30 min. Then the luminescent signal was detected with a Tecan M200 luminometer.

### Evaluation of progressed chondrocyte terminal differentiation following “long term” GSK3β inhibition

- Confocal microscopy analysis of Runx-2 and β-catenin nuclear localization
  Nuclear translocation of pivotal transcription factors (Runx-2 or β-catenin) following LiCl delivery was evaluated in 3 different experiments by confocal microscopy in 4% paraformaldehyde fixed 5 µm micromass sections, essentially as described in^28^. β-catenin translocation was also assessed after SB216763.
- Mineralization assays
  Changes in mineralization following either LiCl or SB216763 were quantified by mean of the Quantichrom DICA-500 assay (Bioassay Systems) as previously described^28^. Micromasses were also scored for mineralized areas by alizarin red staining as previously described^36^.
- Viability assay

The effects on terminal differentiation and viability of chondrocytes were evaluated with the Live & Dead viability cytotoxicity kit (Invitrogen), according to manufacturer’s instructions. Three different experiments of long term GSK3β inhibition were evaluated with NIKON confocal microscope system A1 with a z height of nearly 50 µm, while three additional experiments with micromasses exposed either short term or long term to GSK-3β inhibiting stimuli were analyzed with the ZEISS Light Sheet system. Nuclei were stained with Hoechst 33342.

### MMPs and TIMP-3 quantitative assessment

Supernatants of micromasses exposed to GSK3 inactivating stimuli were used to test the global effect of this treatment on the expression and activity of major collagenases. To this end we used the Human MMP panel 5-PLEX (Biorad) multiplex bead based sandwich immunoassay kit to simultaneously evaluate 5 different MMPs: the three collagenases MMP-1, MMP-8, MMP-13 and the two stromelysins MMP-3 and MMP-10. A further collagenase activator (MMP-14, that is also a collagenase able to cleave collagen 2) was instead analyzed *in situ* with imunohistochemistry (using MAB9181, R&D) coupled with image analysis as previously described^35^ since it corresponds to a membrane type MMP and therefore not released in the supernatant until lately. TIMP-3 was also assessed *in situ* (using MAB973, R&D), since it is the only TIMP to be sequestered to the extracellular matrix^37^.

### Real time PCR

To detect the effects of GSK3β inhibition on global gene reprogramming targeted to ECM remodelling, micromasses underwent RNA extraction with Trizol (Invitrogen), reverse transcription and real time PCR analysis to evaluate change in gene expression of MMPs and TIMPs essentially as described in^20^. Primers were as follows, with details of GenBank entries and primer pairing positions: GAPDH, NM_002046: forward 579–598 and reverse 701–683; MMP-1, NM_002021.3: forward 136–155 and reverse 304–285; MMP-8, transcript variant 1, NM_002424.2: forward 141–160 and reverse 329–310; transcript variant 2, NM_001304441: forward 141–160 and reverse 420–401 and transcript variant 3, NM_001304442: forward 141–160 and reverse 417–398; MMP-13, NM_002427: forward 496–511 and reverse 772–756; MMP-10, NM_002425.2, forward 1278–1298 and reverse 1472–1449; MMP-14, NM_004995: forward 3174–3193 and reverse 3375–3356 and TIMP-3, NM_000362: forward 1193–1210 and reverse 1313–1294.

### Assessment of collagenase activation following “long term” GSK3β inhibition

- Western blotting
  Western blot analysis was performed to detect MMP proteolytic processing. Protein lysates corresponding to half micromass were loaded and processed as described in^28^. Signals were revealed with ECL Select kit (GE Healthcare) as in^20^.
  MMP-13 was detected by a goat polyclonal antiserum (R&D) able to recognize the 60 kDa pro-enzyme, the 50 kDa intermediate active and the 48 kDa finally active form.
  At least 4 different experiments were carried out for each analysis. Anti-GAPDH (clone 6C5, Chemicon–Millipore) served as loading control.
- Collagen zimography
  Supernatants obtained in serum free conditions were tested by zymography^38^ using SDS-PAGE and 10% acrylamide gels containing either gelatin or collagen 1 or 2, to better characterize the type of proteolytic activity. 20 µl supernatant were loaded along with suitable molecular weight markers. Digestion time was 16 hours.
- C1,2C immunohistochemistry

Collagenase activation was also analysed by mean of immunohistochemistry of C1,2C (rabbit polyclonal, IBEX) collagen neoepitopes coupled with image analysis^35^, carried out on samples from three different patients.

### Small interfering RNA mediated GSK3β gene silencing

To confirm the specificity of the effects observed following GSK3β pharmacological inhibition, experiments were also carried out using RNA interference (RNAi), as in^20^.

48 hours after siRNA (either ON-TARGETplus GSK3β or ON-TARGET plus Non-targeting Pool, Dharmacon) delivery, chondrocytes were collected for evaluation of GSK3β knockdown (KD) and for micromass seeding. To confirm the findings obtained with wild type chondrocytes, control siRNA transfected chondrocytes were also stimulated with GSK3 inactivating stimuli at both short or long term.

Immunohistochemistry coupled with image analysis was carried out to detect the effects on selected markers: 8-oxo-dG, MMP-10 (Novus, rabbit polyclonal), MMP-14, TIMP-3 and C1,2C.

Parallel micromass samples were also dedicated for caspase activity analysis.

### Statistics

All experiments were run at least three times or otherwise indicated.

The graphs represent the cumulative analysis of experiments performed with cells from at least three different patients. All data are presented as mean ± standard error of the mean (SEM), analyzed and graphed using GraphPad Prism version 5.00 for Windows (GraphPad Software).

Since comparisons were always carried out in order to compare each GSK3β inhibiting stimulus with the unstimulated condition we did not use statistical tests for multiple groups. Means of groups were compared with Student’s t test as detailed in the legend and considered significant when P < 0.05, with *P < 0.05; **P < 0.01; ***P < 0.001. Two tailed Student’s t test was used throughout. Correlation was tested by Pearson’s r.

## Results

### GSK3β is the prevalent GSK3 isoform in chondrocytes grown in 3-D

When grown in 3-D, chondrocytes recover a phenotype closer to that of articular chondrocytes compared to previous reports^15^. Indeed chondrocytes within 1 week micromasses almost only express the GSK3β isoform (see Supplementary Figure 1). Moreover, GSK3β is the only isoform that undergoes phosphorylation in 1 week micromass, a maturation stage that better corresponds to an healthy articular phenotype. We previously reported that progression from this “healthy articular chondrocyte” phenotype to hypertrophy and terminal differentiation occurs *in vitro* across 1, 2 and 3 weeks maturation^24,25^. It is therefore noteworthy to underline that GSK3α phosphorylation is only evident at 3 weeks maturation, albeit at lower intensity compared to that of GSK3β that reaches its maximal levels at this maturation stage. Our 3-D model allows us to confirm that terminal differentiation is characterized by the highest level of GSK3 phosphorylation of both its β and α isoform, in keeping with previously reported information of GSK3 role in differentiation progression^9^ (Supplementary Figure 1: S1)

### GSK3β inhibition in chondrocytes affects mitochondria and determines ROS production

We recently showed that GSK3β inhibition in exponentially growing chondrocytes elicits mitochondria activation and ROS production^20^. Therefore, we evaluated the effect of “short term” GSK3β inhibition in 3-D cultures since these settings more closely reproduce the *in vivo* situation with condrocytes surrounded by their native ECM. We tested the extent of mitochondrial involvement that represents an early event downstream GSK3β inhibition. Altered mitochondrial function was assessed by change of mitochondrial membrane potential, production of ROS, accumulation of 8-oxo-dG adducts.

**Figure 1:**
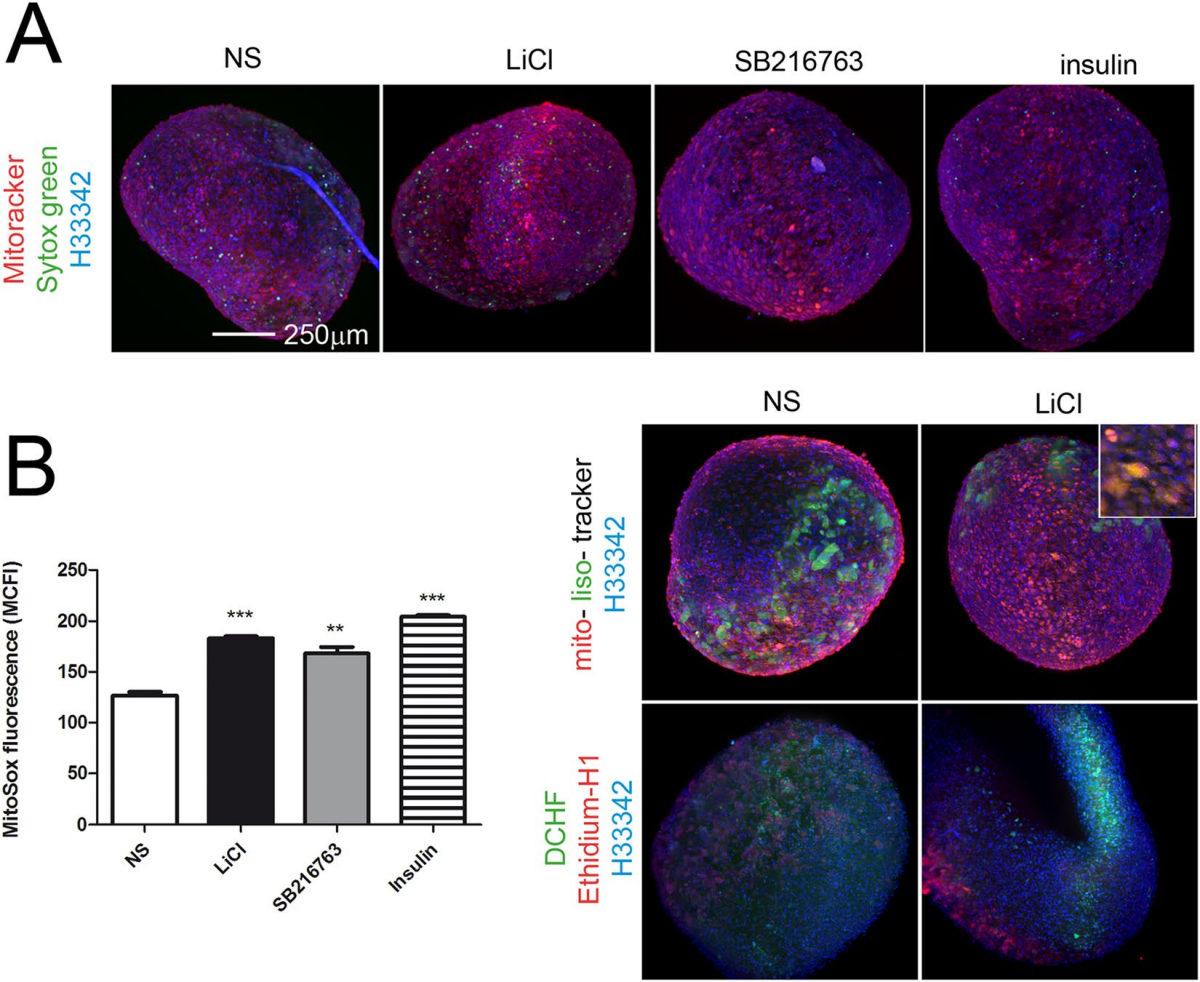
Short term GSK3 inhibition in chondrocytes cultured in 3-D affects mitochondrial functions as evidenced by live sample imaging. (**A**) GSK3β inhibiting stimuli led to increased MitoTracker Orange CMTMRos signal suggestive of increased mitochondrial membrane potential and ROS production (a representative experiments out of three performed; bar:250 µm). (**B**) Left graph, Flow cytometry analysis indicates that GSK3β inhibiting stimuli significantly increased superoxide production, as assessed by the MitoSox probe (a representative experiments out of two performed, with each condition performed in triplicate and compared to basal condition by mean of an unpaired Student’s t test). **P < 0.01; ***P < 0.001. Right images: following LiCl treatment, the ROS damaging effect on mitochondria led to increased mitophagy, as assessed by overlapped MitoTracker Orange CMTMRos and LysoTracker Green signal (Upper images). See the higher magnification inset. The increased ROS accumulation was confirmed by increased DCHF-DA staining (Lower images).

Light Sheet imaging of micromasses treated short term (16 hours) with GSK3β inactivating stimuli showed markedly increased signal of MitoTracker (Fig. 1A), a probe that has been shown to sensibly and reliably evidence changes in transmembrane potential as well as increased ROS generation^29^. Flow cytometric analysis of cells obtained by mild enzymatic digestion indicated that these stimuli caused a markedly increased MitoSox fluorescence and therefore an increased superoxide production, also confirmed by a stronger DCHF signal (Fig. 1B).

**Figure 2:**
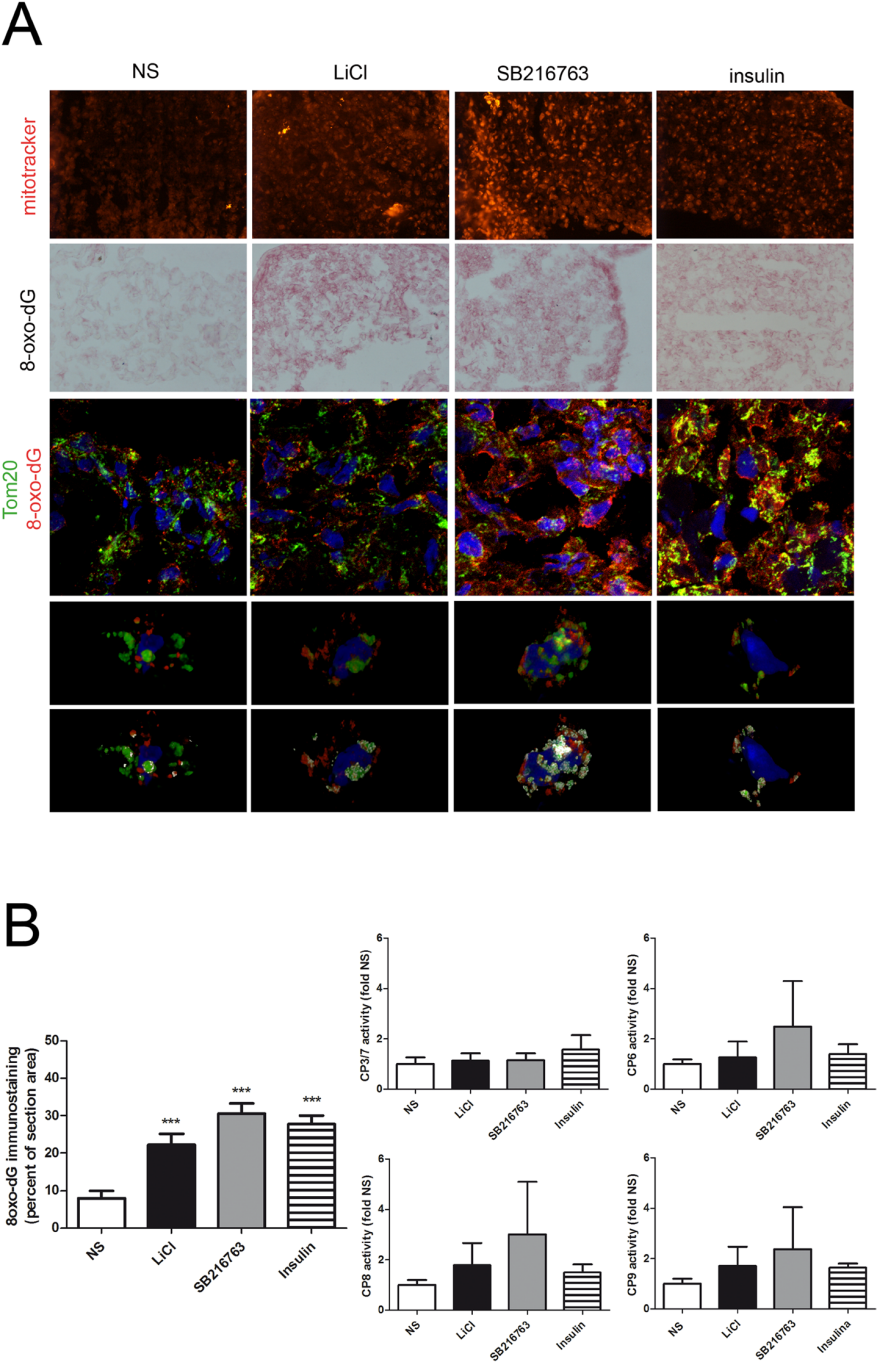
Short term GSK3 inhibition in chondrocytes cultured in 3-D leads to mitochondrial DNA damage as evidenced by in depth analyses. (**A**) First row (magnification 200x): staining of micromass sections confirmed the accumulation of proteins stainable by MitoTracker following GSK3β inhibiting stimuli. Second row (magnification 200x): GSK3β inhibiting stimuli led to increased 8-oxo-dG, an oxidative damage marker. Third row (magnification 600x): accumulation of 8-oxo-dG occurs specifically in mitochondrial DNA: confocal analysis demonstrated the lack of signal in genomic DNA and 8-oxo-dG localization exclusively in the mitochondria, overlapping with the mitochondrial marker Tom20. Fourth and fifth rows are enlargements from correspondent confocal pictures above: quantitative comparison of the intensity of colocalized pixel (filtering the events with fluorescence intensity close to the bisector that were rendered in white) yielded a significantly higher colocalized signal of Tom20 and 8-oxo-dG in all the samples. (**B**) GSK3β inhibiting stimuli led to statistically significant increased amount of 8-oxo-dG (according to cumulative image analyses of experiments performed in triplicate with cells from three different patients, n = 9, and compared to basal condition by mean of an unpaired Student’s t test,***P < 0.001) and to correlated increased activity of caspase 6, 8 and 9 (n = 7). Graphs show caspase 3/7, 6, 8 and 9 activity normalized (Fold change) to the level of control sample.

MitoTracker staining applied to micromass sections confirmed a higher staining with GSK3β inactivating stimuli, particularly in samples treated with SB216763 (Fig. 2A). As also previously shown in monolayer, we found evidence of mitochondrial-nuclear translocation of proteins selectively stained by MitoTracker. This has been previously explained as due to heat shock proteins functioning as chaperones that migrate to the nucleus bound to antiapoptotic proteins^39^, and therefore expression of compensatory activities of the cells to protect themselves from oxidative stress following GSK3β inhibition.

The increased ROS production led to mitochondrial damage that activates mitophagy (Fig. 1B) as a compensatory, yet ineffective mechanism, as judged on the basis of a significantly increased 8-oxo-dG signal following the GSK3β inactivating stimuli (Fig. 2A and B). Double fluorescence analysis staining confirmed the higher amount of 8-oxo-dG in the mitochondria. Colocalization was investigated as described in^40,41^. A 3-D reconstruction of single cells showed that 8-oxo-dG signal was exclusively localized in the mitochondrial and not in the genomic DNA.

Moreover, a quantitative comparison of the intensity of colocalized pixels (filtering the events with fluorescence intensity close to the bisector that were rendered in white in lower images of Fig. 2A) yielded a significantly higher colocalized signal of Tom20 and 8-oxo-dG following all GSK3β inhibiting stimuli (mean ± SD: NS = 0.33 ± 0.01; LiCl = 0.55 ± 0.10; SB216763 = 0.88 ± 0.09; insulin = 0.63 ± 0.04; Student’s t test, p < 0.05). To confirm that 8-oxo-dG was not localized to the genomic DNA a further analysis was undertaken: for each condition three distinct confocal optical sections were taken, at different Z-height, and nuclei were confirmed free of 8-oxo-dG signal (Supplementary Figure 2: S2). Figure S2 reports details of the cells presented in the high magnification (600×) pictures in bottom rows of Fig. 2A.

Following oxidative stress and mitochondrial impairment, the involvement of apoptotic pathways has been investigated. Evaluation of caspase activation carried out on 7 different patients indicated a minor role for caspase 3/7 compared to caspase 6 and a concurrent activation of caspase 9 (the caspase activated after mitochondrial stress) and of caspase 8. The analysis was affected by a certain degree of variability, possibly due to the different extent of chondrocyte viability in the 3-D constructs. Yet, the cumulative analysis of the activities of the four caspases (21 samples) showed a very high level of correlation among CP6, 8 and 9 (CP8 vs CP9 r = 0.9919; CP8 vs CP6 r = 0.9863; CP9 vs CP6 r = 0.9817) suggesting their interdependent activation downstream GSK3β inhibition. Although not significantly different, the GSK3β specific inhibitor SB216763 was the more effective stimulus for caspase activation (see Fig. 2B). The lower level of caspase activation exerted by LiCl or insulin was likely due to their stimulatory effect on the PI3K/Akt pathway^42^ known to be able to reduce caspase activation^43,44^.

### GSK3β inhibition affects chondrogenesis progression to terminal differentiation and viability in 3-D cultures

3-D cultures were used to evaluate the effect of “long term” GSK3β inhibition on chondrocyte maturation across the chondrogenesis process to terminal differentiation.

**Figure 3:**
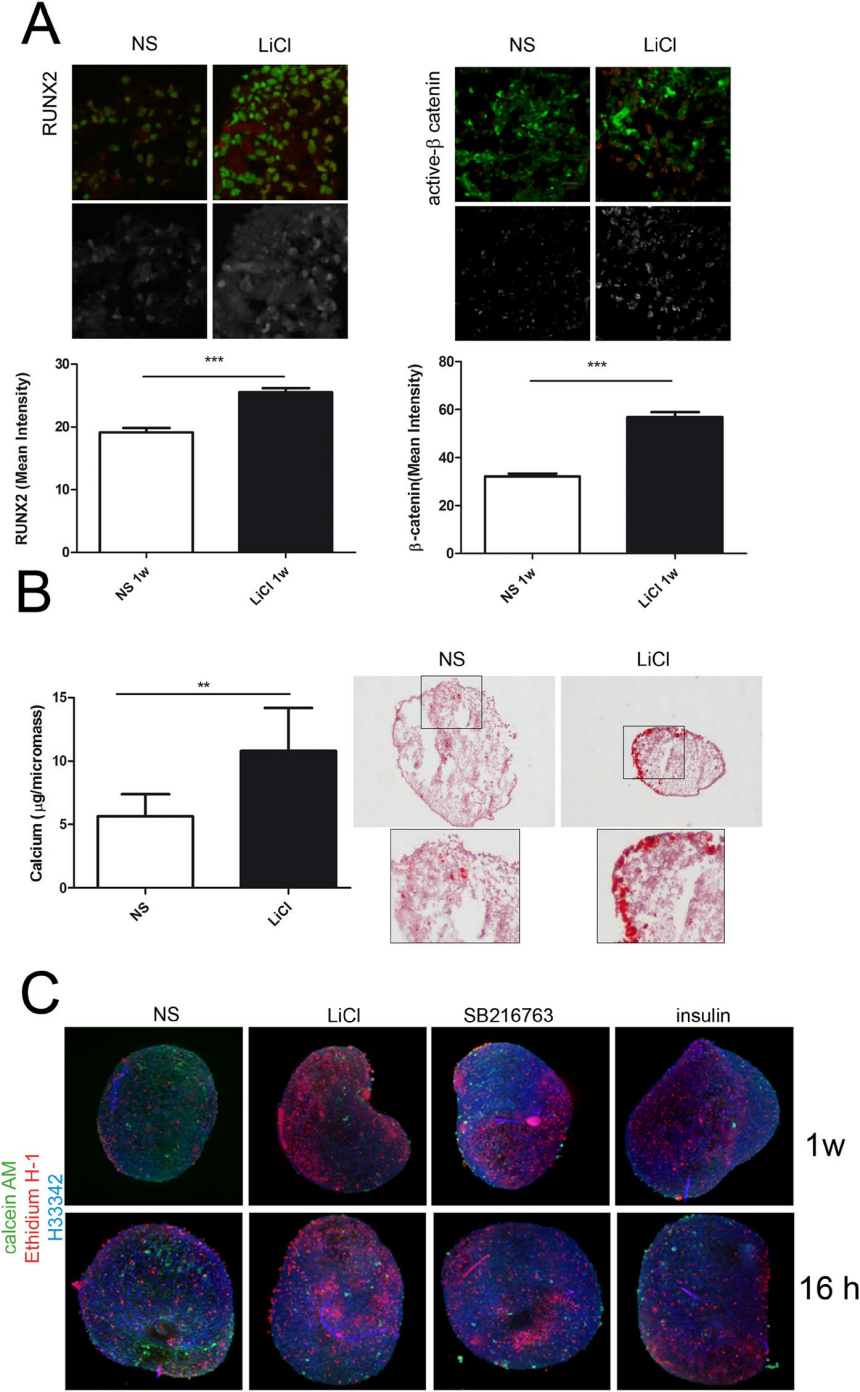
GSK3 inhibition affects viability and chondrogenesis progression to terminal differentiation in 3-D cultures. (**A**) “long term” treatment with LiCl increased nuclear translocation of transcription factors directing chondrogenesis: representative images of merged signals of nuclei (stained with Sybr Green) and either Runx-2 (left images) or active β-catenin (right images) stained with TRITC or DYLight 647 conjugate anti-primary antibodies. Graphs report the mean nuclear intensity of immunofluorescent staining in several 60x fields taken from sections of micromasses at 1 week of culture. The means are relative to several hundreds of different cell nuclei (Runx-2: NS, 365; LiCl, 637. β-catenin: NS, 2137; LiCl, 1441) as evaluated by an unpaired Student’s t test. ***P < 0.001 (**B**) LiCl treatment leads to a statistically significant increased calcium deposition (n = 10, paired Student’s t test, *P < 0.05) as quantified by Quantichrom DICA-500 assay and confirmed by alizarin-red staining (right images). (**C**) The GSK3β inhibiting stimuli led to increased cell death as detected by live sample imaging with live and dead staining (calcein AM and ethidium homodimer) and Light Sheet detection. Nuclear counterstaining was performed with Hoechst 33342. A representative example out of three different experiments, each performed with cells derived from different patients.

Confocal analysis indicated that nuclear localization of the hypertrophy marker and regulator Runx-2 was much higher in 5 mM LiCl treated micromasses. This led to increased nuclear translocation and therefore transcriptional activation of β-catenin. Comparable results were obtained with SB216763 (Supplementary Figure 3). Graphs in Fig. 3A, reporting the cumulative intensity of several hundreds of cells, indicated statistically significant higher nuclear signal for both these transcription factors pivotal in chondrocyte maturation. This was in keeping with statistically significantly higher mineralization content of 1 week 5 mM LiCl stimulated micromasses, as evaluated by the DICA-500 assay (n = 10), and confirmed by alizarin red staining (Fig. 3B). Instead, mineralization was not affected by SB216763 (n = 7, Supplementary Figure 4). The effect of GSK3β inhibition in prompting terminal differentiation was also confirmed by live and dead analysis of 1 week micromasses. GSK3β inhibition achieved by 5 mM LiCl, 10 µM SB216763 or 33 nM insulin markedly affected cell viability as indicated by the higher frequency of dead cells that was evident following both short and long term treatment. Figure 3C shows one representative example out of three different experiments performed.

### GSK3β inhibition *“per se”* is sufficient to concertedly regulate transcription, translation and post-translational processing and activity of collagenases

**Figure 4:**
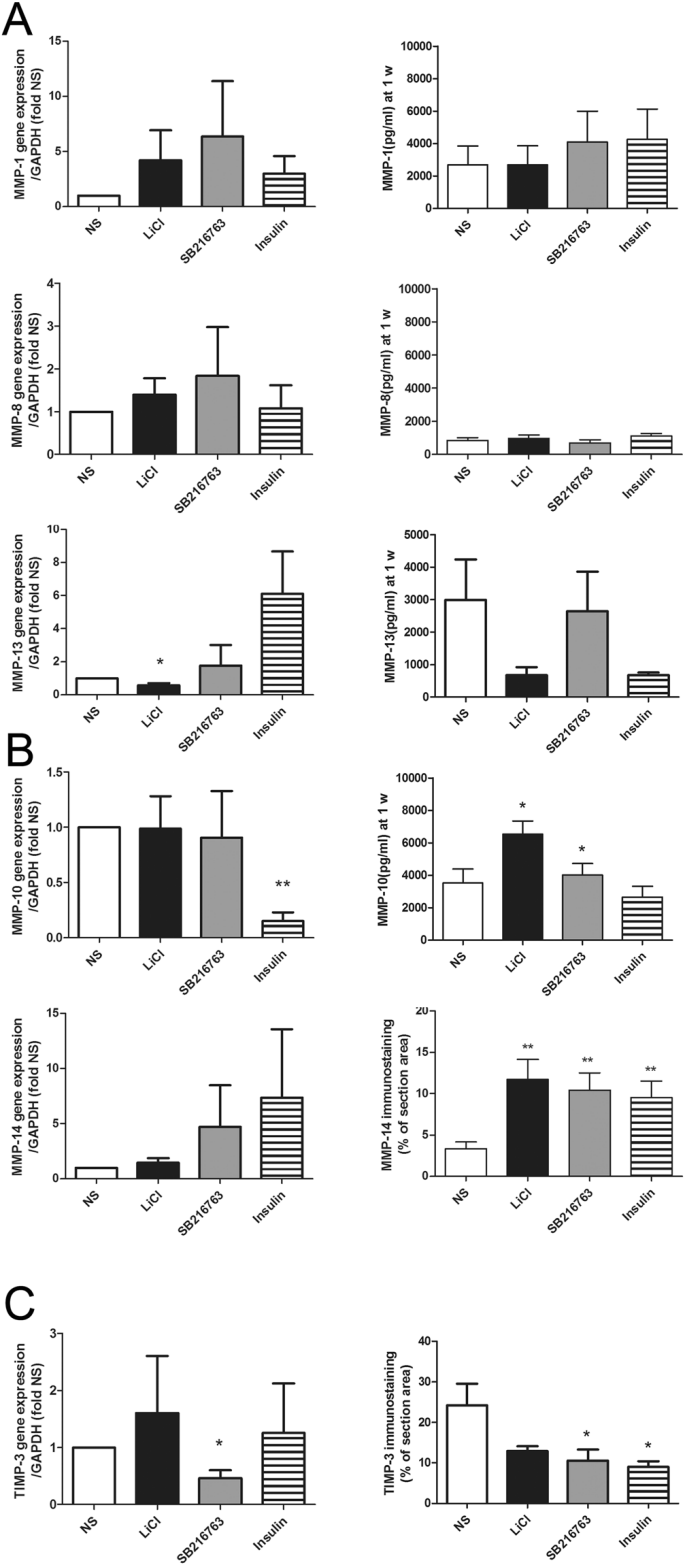
Long term GSK3 inhibition *per se* is sufficient to concertedly regulate transcription and translation of collagenases. **(A**) Effects of GSK3β inhibiting stimuli on mRNA (as assessed by real time PCR, left graphs; n = 6) and protein (as assessed by multiplex bead based sandwich immunoassay kits; right graphs, n = 7) expression of major collagenases (MMP-1, MMP-8 and MMP-13). (**B**) Effects of GSK3β inhibiting stimuli on mRNA (left graphs; n = 6) and protein expression of major collagenase activators (right graphs; MMP-10, n = 7 and MMP-14, n = 3, the latter investigated *in situ* by image analysis of immunohistochemistry detection and expressed as % of section area, i.e. the percentage of staining area relative to the area of the tissue). (**C**) Effects of GSK3β inhibiting stimuli on mRNA (left graphs) and protein (right graphs) expression of TIMP-3, the pivotal inhibitor of both collagenases and aggrecanases, as assessed by image analysis *in situ* (n = 3 different patients) since it is the only TIMP with a binding domain to ECM. *P < 0.05, **P < 0.01.

The regulation of ECM remodelling downstream GSK3β inhibition was investigated evaluating at both RNA (Fig. 4, left graphs) and protein (Fig. 4, right graphs) level the effect on major collagenases (Fig. 4A MMP-1, 8 and 13) and on activators (Fig. 4B MMP-10 and 14) and major inhibitor (Fig. 4C TIMP-3) of their activity. No evident transcriptional effect was appreciated on collagenases, but a reduction of MMP-13 in LiCl treated samples (Fig. 4A).

However, a strong activation of both MMP-10 and MMP-14 protein expression (the former evaluated in the supernatants and the latter by means of immunohistochemistry performed on three different experiments) was observed following both LiCl and SB216763 (Fig. 4B). MMP-10^45^ and MMP-14^46,47^ are pivotal activators of MMP-13 in OA tissue and at the same time members of the senescence associated secretory phenotype^48^. On the other hand, this was paralleled by a consistently reduced expression of TIMP-3 (Fig. 4C), the major MMP inhibitor in chondrocytes, the only one capable of inhibiting both aggrecanases and MMPs, and whose knockdown has been previously recognized as responsible for OA development^49^. Notably, SB216763, a GSK3β specific inhibitor^50^, was effective in inhibiting both TIMP-3 transcription and translation. GSK3β inhibition did not affect MMP-3 levels in the supernatants (data not shown).

### GSK3β inhibition impacts on the regulation of collagenase activity

**Figure 5:**
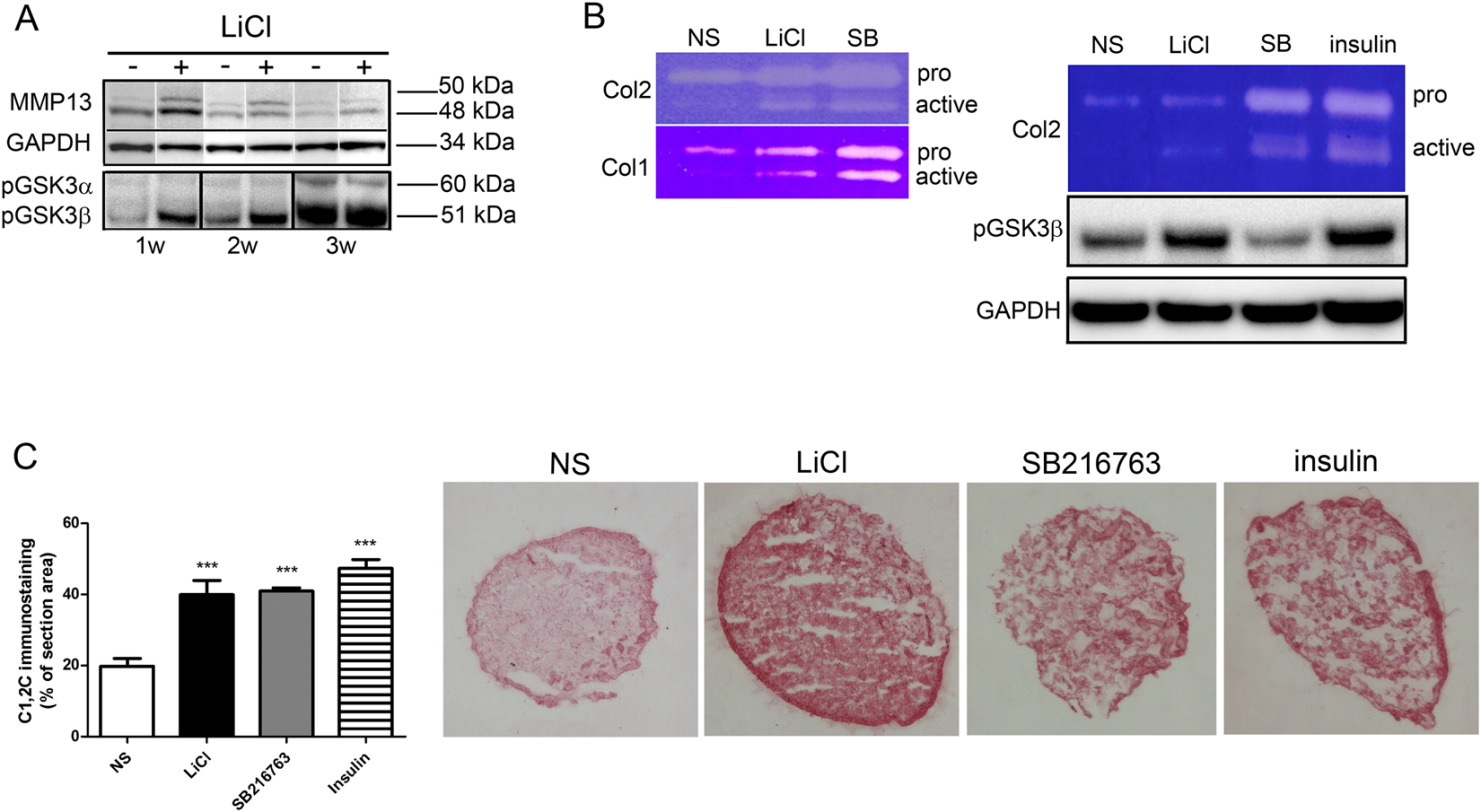
GSK3 inhibition impacts on collagenase post-translational processing and on the regulation of collagenase activity. **(A**) The GSK3β inhibiting stimulus LiCl led to procollagenase proteolytic processing of MMP-13 during 3-D culture maturation (1w = 1 week, 2w = 2 weeks, 3w = 3 weeks). Western blot of micromass lysates showed increased evidence of the 50 kDa intermediate form and of the 48 kDa finally active form (left panel); this was matched by increased phosphorylation of the GSK3β isoform at 1 and 2 weeks. (**B**) Zymography of the culture supernatants confirmed an increased collagenolytic activity on both Col1 and Col2 substrates (left side), with an increase in the proenzymatic and active form. Insulin and SB216763 showed the strongest collagenolytic activity on Col2 (right image, SB:SB216763). Again, the increased collagenolytic activity is accompanied by an increased GSK3β phosphorylation. (**C**) The GSK3β inhibiting stimuli induce increased accumulation of the collagen neoepitope C1,2C *in situ*, as confirmed by immunohistochemistry detection and image analysis (n = 9: triplicate micromasses derived from three different patients. Comparisons with basal condition were performed by an unpaired Student’s t test). ***P < 0.001. Pictures relative to a representative patient out of the three analyzed.

As shown above, GSK3β inhibiting stimuli determined pleiotropic effects on MMPs and TIMPs, and therefore to test the final effect on collagenase activity we investigated the proteolytic processing of MMP-13, the pivotal collagenase in OA^51^, within the 3-D construct. The representative example in Fig. 5A shows that across micromass maturation, LiCl addition leads to an increase of the 50 kDa intermediate active form and of the 48 kDa finally active form. The increased MMP-13 proteolytic processing following LiCl mediated GSK3β inhibition was maximally evident at 1 week but also maintained at further micromass maturation stages (Fig. 5A), that more closely correspond to terminal differentiation^25^. However, only at 1 week the increased MMP-13 proteolytic processing was markedly associated to a strong increase in GSK3β phosphorylation in LiCl stimulated samples. GSK3α phosphorylation was only evident at 3 weeks but independently of the stimuli and rather indicating a progression in differentiation (Supplementary Figure 1: S1).

To investigate the collagenase activity following GSK3β inhibition, serum starved supernatants were tested in zymography, in presence of Col2 as substrate. A representative example out of 4 different experiments is shown in Fig. 5B. GSK3β inhibiting stimuli led to a markedly increased collagenase activity as evidenced by increased signal of both the proenzyme and of the proteolytically processed form. Zymographic activity of supernatants tested on gelatin or Col1 gels showed a similar pattern, suggesting that GSK3β inhibition leads to a marked increase of the released enzymatic activity with enhanced proteolysis of different ECM components and independently of substrate specificity^52^. In 1 week micromasses, the increased collagenolytic activity paralleled that of GSK3β inactivation via phosphorylation, with the exception of the use of SB216763 that specifically inhibits GSK3 in an ATP dependent manner and without phosphorylation.

The increased collagenolytic activity following GSK3β inhibiting stimuli delivered to 1 week micromasses was also confirmed *in situ* by a markedly increased C1,2C staining, as shown in Fig. 5C.

### The effects of GSK3 inhibitors are reproduced by GSK3β specific knockdown

To assess the specificity of the effects observed downstream GSK3 inactivating stimuli, we carried out specific silencing of GSK3β, reported to be the prevalent form in articular chondrocytes^9^. Chondrocytes transfected with control vector were treated short term or long term with the GSK3 inactivating stimuli to compare the pharmacologic inhibition and the RNAi approach.

**Figure 6:**
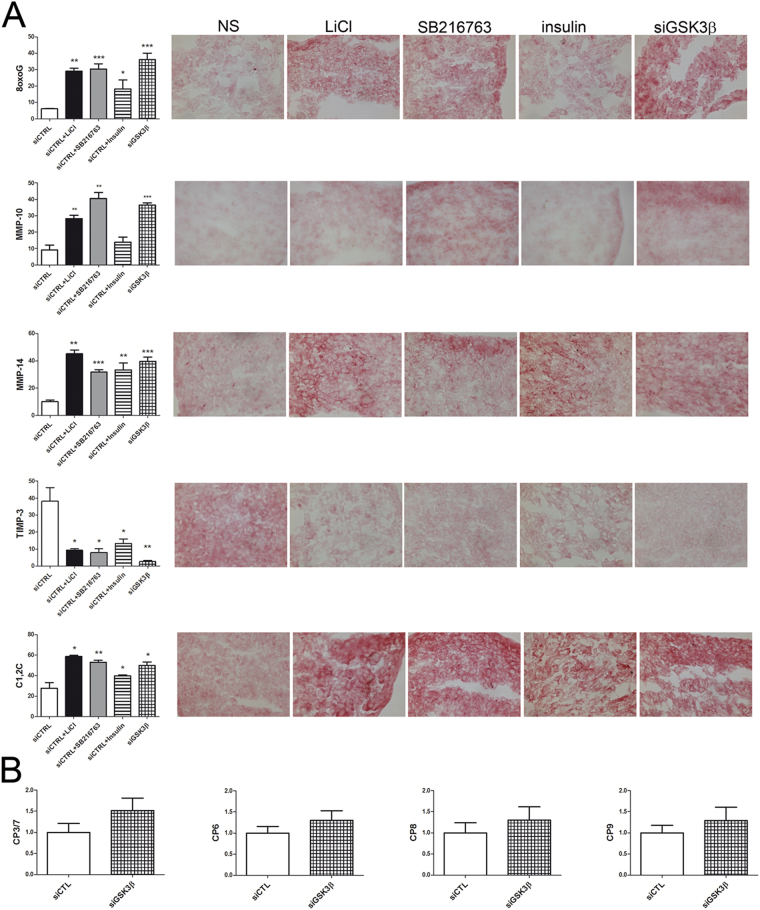
The effects of GSK3 inhibitors are reproduced by GSK3β specific knockdown. (**A**) To confirm the GSK3β specificity of the effects observed downstream GSK3 inhibiting stimuli, chondrocytes were transduced with control (CTRL) or GSK3β siRNA. In particular, siCTRL micromasses underwent GSK3 inhibiting treatment either short or long term, and the effects were compared with those observed in micromasses seeded with siGSK3β chondrocytes. siGSK3β reproduced all the effects observed downstream GSK3β inhibiting stimuli: increased accumulation of 8-oxo-dG adducts at 16 hours and increased activation (higher level of MMP-10 and MMP-14) and decreased inhibition (lower TIMP-3) of collagenase activity at 1 week, thus resulting in higher amount of C1,2C neoepitopes. Graphs on the left report image analysis of immunohistochemistry experiments. The data shown are representative of one out of two performed. *P < 0.05, **P < 0.01, ***P < 0.001. (**B**) Moreover, siGSK3β led to increased activation of initiator (caspase 8 and 9) and effector (caspase 3/7 and 6) caspases as shown by cumulative (n = 2) analysis of increased activity of these enzymes in 1 week micromass lysates and expressed as fold change compared to the control siRNA.

Figure 6 shows the results of one representative experiment and the cumulative image analysis of 8-oxo-dG, MMP-10, MMP-14, TIMP-3, C1,2C and caspase activity.

Delivery of the GSK3 inactivating stimuli confirmed the effects observed with wild type chondrocytes, and GSK3β specific silencing reproduced the effects on mitochondrial DNA damage, increase in collagenase activators (MMP-10 and MMP-14) and decrease in the major collagenase inhibitor (TIMP-3) thus leading to increased C1,2C accumulation (Fig. 6A).

Micromass lysates were tested for the activity of major caspases. Specific silencing of GSK3β led to increased activity of all caspases tested (Fig. 6B).

## Discussion

Litherland and co-workers had recently pointed out that GSK3 inhibition, rather than being protective as previously suggested^18^ worsens murine OA^15^. Moreover, GSK3 inhibition is an event placed downstream many inflammatory stimuli^15^ or growth factors that signals via PI3K/Akt to inhibit GSK3 such as insulin, increased in insulin resistance and recognized as a major determinant of metabolic OA^53^ and mitochondrial dysfunction^54^. Noteworthy, fractionation studies indicated that GSK3 localizes in the cytosolic, mitochondrial and nuclear fraction, with different proportion depending on the cell type^19^. Inhibition of “mitochondrial GSK3” is responsible for increased ROS production and downstream effects on cellular senescence, as the result of mitochondrial complex IV defect^19^.

Mitochondrial pathology has been recently recognized as having a pivotal role in OA^55,56^ since ROS produced by dysfunctional mitochondria are able to boost cellular signalling and matrix catabolism^55^ thus leading to tissue derangement.

We had already shown that mitochondrial activation and ROS production occur after GSK3β inhibition in primary chondrocytes cultured in “monolayer”, reproducing the phenotype observed in cartilage of obese patients where GSK3β phosphorylation overlaps with mitochondrial DNA damage, hypertrophy and senescence^20^. Concerning articular cartilage, data derived from the different phenotypes of global GSK3-α or β knockout indicate a more pivotal role for GSK3β that is also selectively expressed in articular chondrocytes^9,14^. To approach *in vitro* the closest as possible the phenotype of articular chondrocytes *in vivo* we selected a 3-D culture model (micromass or pellet cultures)^22^ that more closely mimic the complex scenario of chondrocytes embedded in their native ECM^57^. In micromasses, chondrocytes recover in 1 week time a correct “healthy articular chondrocyte” phenotype. Indeed, the analyses carried out in these settings confirmed that the β isoform accounts for most of GSK3 expression and phosphorylation.

Then we tested the effect of GSK3β inhibition on chondrocyte differentiation using 3-D cultures of chondrocytes. GSK3β inhibition was achieved by mean of both pharmacological and RNAi approach. We provide evidence that GSK3β inhibition *“per se”* achieved with different stimuli and independently of other upstream inflammatory stimuli is sufficient to elicit mitochondrial activation, superoxide production and oxidative damage (8-oxo-dG staining) to mitochondrial DNA in 3-D culture of primary chondrocytes. This results in activation of initiator caspases 8 and 9 and effector caspase 6 and 3/7. These events are sufficient to increase nuclear localization of transcription factors (Runx-2, β-catenin) responsible for enhanced chondrocyte differentiation towards “endochondral ossification”. Indeed, we described an increased calcium deposition and “terminal differentiation” of chondrocytes as indicated by live and dead experiments carried out with Light Sheet technology, an advanced method of fluorescence microscopy, specifically developed for 3-D structure mapping of large samples^58^, a challenge that could allow to drive more relevant conclusions compared to conventional studies carried out in monolayer. These experiments indicate reduced viability at both “short term” and “long term” GSK3β inhibition.

We also dissected the mechanisms whereby GSK3β inhibition leads to ECM remodelling. Overall, GSK3β inhibition achieved with either pharmacological or RNAi approach consistently impacts on the regulation of collagenase activity as shown by zymography or accumulation of the C1,2C neoepitope, a bioactive peptide able to further boost chondrocyte progression towards hypertrophy and endochondral ossification^2^. Noteworthy, at the same time GSK3β inhibition increases the protein expression of major collagenase activators (MMP-10 and MMP-14) and reduces the expression of TIMP-3, the only one inhibitor that is effective on both aggrecanases and MMPs. Notably, functional genomic studies in KO mice had previously indicated the role of TIMP-3 in OA progression^49^.

The concerted effect on collagenase activity observed with pharmacological inhibition of GSK3β using either specific (SB216763) or aspecific drugs (insulin and lithium chloride, that can be considered as an insulin mimicker) were fully reproduced by a RNAi approach, thus confirming that GSK3β is one of the molecular constraints that prevent the progression of articular chondrocytes towards terminal differentiation.

In conclusion exploiting 3-D culture of primary chondrocytes we here demonstrate that GSK3β inhibition leads to a highly catabolic shift in protein expression and posttranslational processing, that through accumulation of bioactive collagen peptides boosts chondrocyte progression towards terminal differentiation, further sustained by accumulation of mitochondrial damage.

## Electronic supplementary material

Supplementary Figures
