# Supplementary material for: Glycogen Synthase Kinase-3β Inhibition Links Mitochondrial Dysfunction, Extracellular Matrix Remodelling and Terminal Differentiation in Chondrocytes
Source: Sci Rep. 2017 Sep 21;7:12059. doi: 10.1038/s41598-017-12129-5 (PMC5608843; doi:10.1038/s41598-017-12129-5)
Supplement: Supplementary file 1 — Supplementary Figures [file 41598_2017_12129_MOESM1_ESM.pdf]

Supplementary Figures

GLYCOGEN SYNTHASE KINASE-3β INHIBITION LINKS MITOCHONDRIAL DYSFUNCTION, EXTRACELLULAR MATRIX REMODELING AND TERMINAL DIFFERENTIATION IN CHONDROCYTES

Serena Guidotti; Manuela Minguzzi; Daniela Platano; Spartaco Santi; Giovanni Trisolino; Giuseppe Filardo; Erminia Mariani; Rosa Maria Borzì

S1

A (Monolayer)

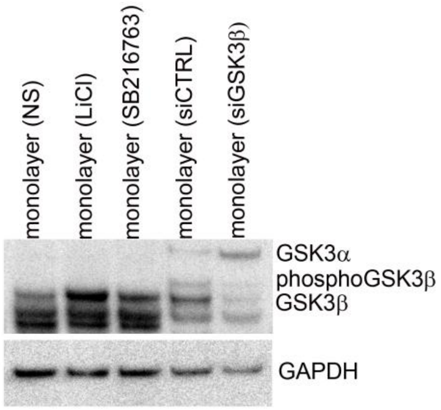

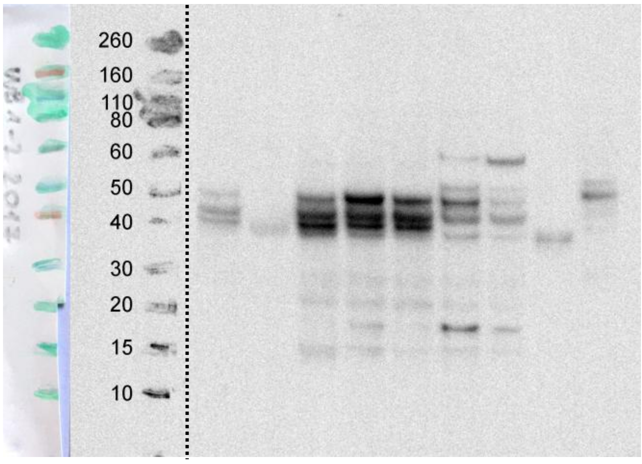

B (Micromasses at 1 week maturation)

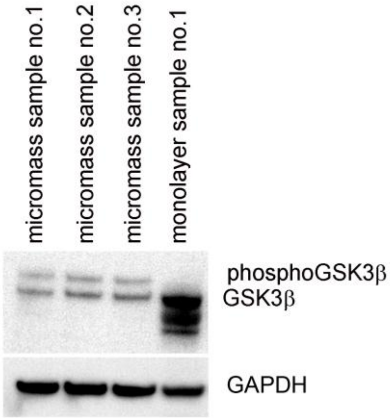

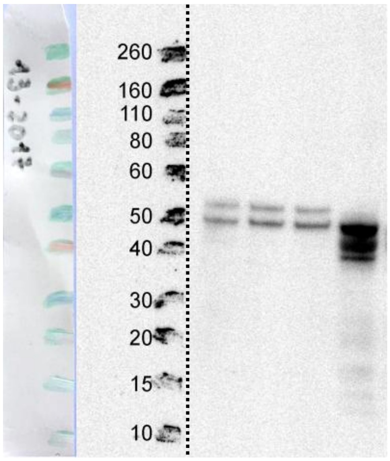

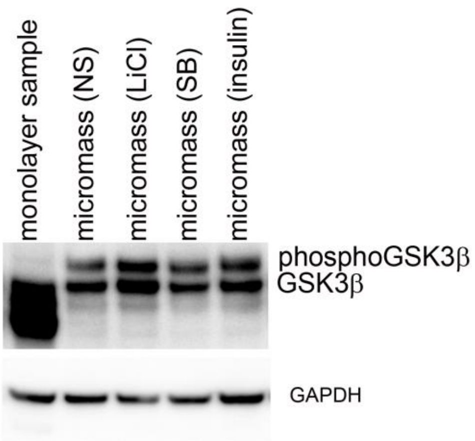

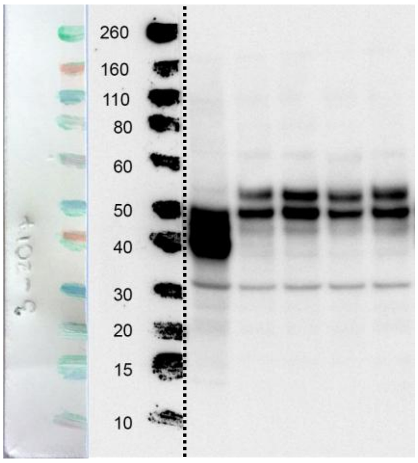

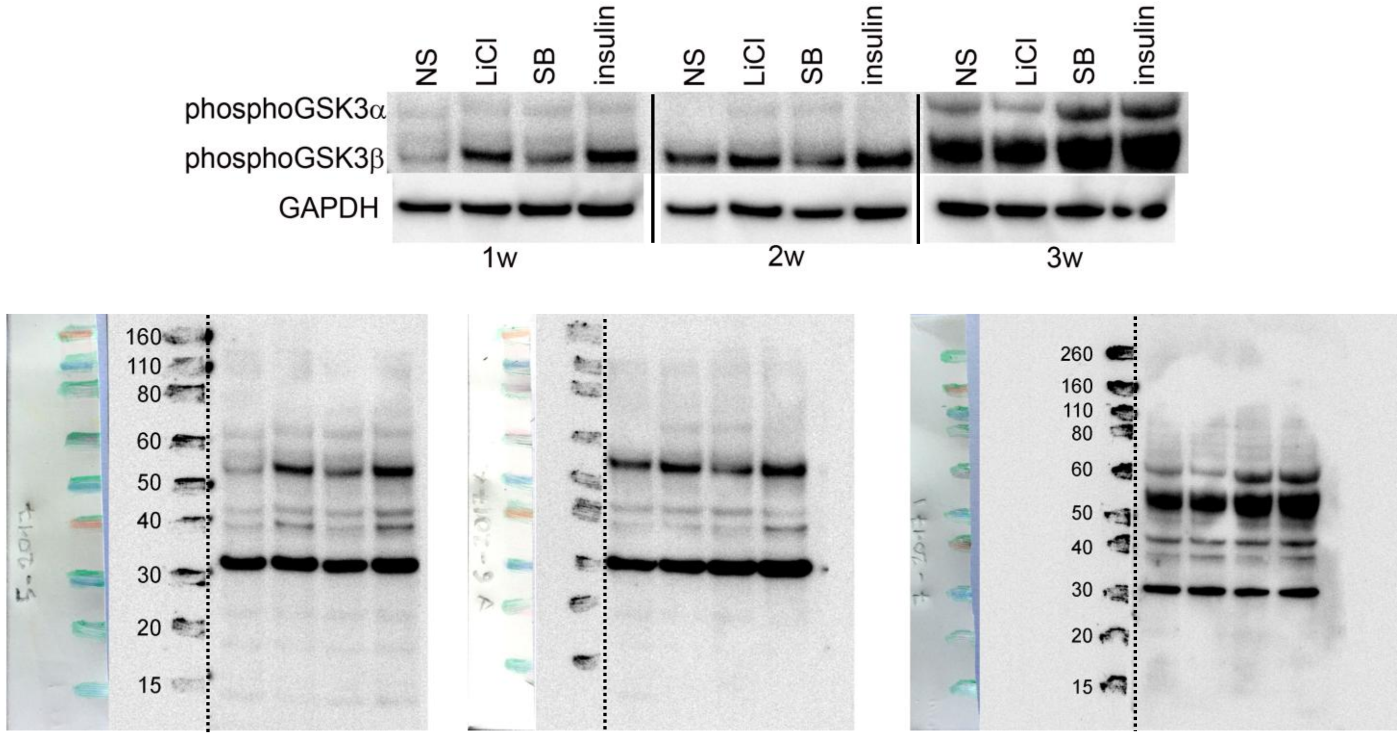

Supplementary Fig. S1. Insets with sample details and original western blots showing expression of GSK3α and β phosphorylated and non phosphorylated forms in chondrocytes cultured in monolayer (A) and micromasses (B). To detect GSK3α and β we exploited western blot analysis using different antibodies (detailed in the paper and in the table) and conditions to maximize protein resolution and separation in the 50-60 kDa range (NuPAGE Novex 4-12% Bis-Tris Gel run with 1x MES running buffer). Moreover, we used Carestream Molecular Imaging Software to accurately assess the molecular weight of the bands exploiting a proper molecular weight marker (Novex Sharp Pre-stained Protein Standard, https://www.thermofisher.com/order/catalog/product/LC5800). To assess the molecular weight of western blot stained bands the pre-stained bands of the marker were highlighted by mean of a Glow Writer pen (http://divbio.com/glow-writerpen.aspx). Images show pictures of the pre-stained molecular weight markers run in each gel along with their corresponding chemiluminescent pattern. We carried out an accurate automatic assessment of the molecular weight of GSK3α and β unphosphorylated and phosphorylated forms and found the following:

Number of assessments	**GSK3beta (n=12)** ***(SCBT#7291, CST#9315)***	**phosphoGSK3beta (n=13)** ***(SCBT#7291, CST#9331, CST#5558)***	**GSK3alfa (n=1)** ***(SCBT#7291)***	**phosphoGSK3alfa (n=2)** ***(CST#9331)***
Mean of molecular weight assessed	**47.88**	**51.38**	**57**	**59.83**
Standard deviation	**1.30**	**1.68**		**1.17**

A. Probing with the validated Santa Cruz Antibody #7291 (http://www.proteinatlas.org/ENSG00000082701-GSK3B/antibody) indicated that GSK3β is the only GSK3 isoform detectable in chondrocytes cultured in monolayer in our experimental settings. GSK3α is only detectable in chondrocytes treated with either control or GSK3β siRNA and increased in the latter in keeping with previous literature findings (Gillespie, J. R. *et al.* Deletion of glycogen synthase kinase-3beta in cartilage results in up-regulation of glycogen synthase kinase-3alpha protein expression. *Endocrinology* **152**, 1755-1766, doi:10.1210/en.2010-1412, reference n. 9 of the manuscript).
The finding of GSK3β being the prevalent form expressed is in keeping with the information available in Proteinatlas.org: the expression of GSK3β (http://www.proteinatlas.org/ENSG00000082701-GSK3B/cell#human) is much more constitutive than that of GSK3α (http://www.proteinatlas.org/ENSG00000105723-GSK3A/cell). #7291 antibody allows to appreciate both the phosphorylated and the non-phosphorylated form, with the former migrating slightly slower than the other.

B. Probing with the validated Santa Cruz Antibody #7291 (http://www.proteinatlas.org/ENSG00000082701-GSK3B/antibody) indicated that GSK3β is the only GSK3 isoform detectable in chondrocytes cultured in micromasses in our experimental settings using cells of four different patients. The upper blot reports results obtained with samples of 1 week micromasses established with cells derived from three different patients, besides one sample of monolayer chondrocytes. The lower blot shows data obtained with micromasses established with cells of another patient following GSK3β inhibiting stimuli. The relative expression of GSK3β is much higher in monolayer compared to micromass chondrocytes. The prevalent form is the non phosphorylated GSK3β, that is also the only one evident in monolayer. No bands are appreciable running at about 60 kDa, the molecular weight corresponding to GSK3α in our conditions.
In the following western blot the phosphorylation of both GSK3β and GSK3α after GSK3 inactivating stimuli was investigated using the Cell Signaling antibody #9331. Shown are the results obtained with samples at 1, 2 and 3 weeks maturation. The pattern of phosphorylation obtained in 1 week micromasses indicated that, in this setting best mimicking that of healthy articular chondrocytes, only the GSK3β isoform undergoes phosphorylation. All the experiments (but that shown in Figure 5A) presented in the manuscript have been carried out in 1 week micromasses. GSK3α undergoes phosphorylation only at 3 weeks maturation, but still, the phosphorylation of GSK3β is much stronger. Collectively these results emphasize the role of GSK3 inactivation in terminal differentiation and the major role of GSK3β.

In both **A** and **B** the black solid vertical lines separate bands that were not adjacent as derived from the different full blots shown in the lower part of the figure.
The dotted black vertical lines delineate the markers that were run in each gel and highlighted by mean of a Glow Writer pen before luminescent detection of the signals. All molecular weight markers run adjacent to the bands shown beside them, but after transfer and before membrane probing were cut and re-aligned for the detection.

S2

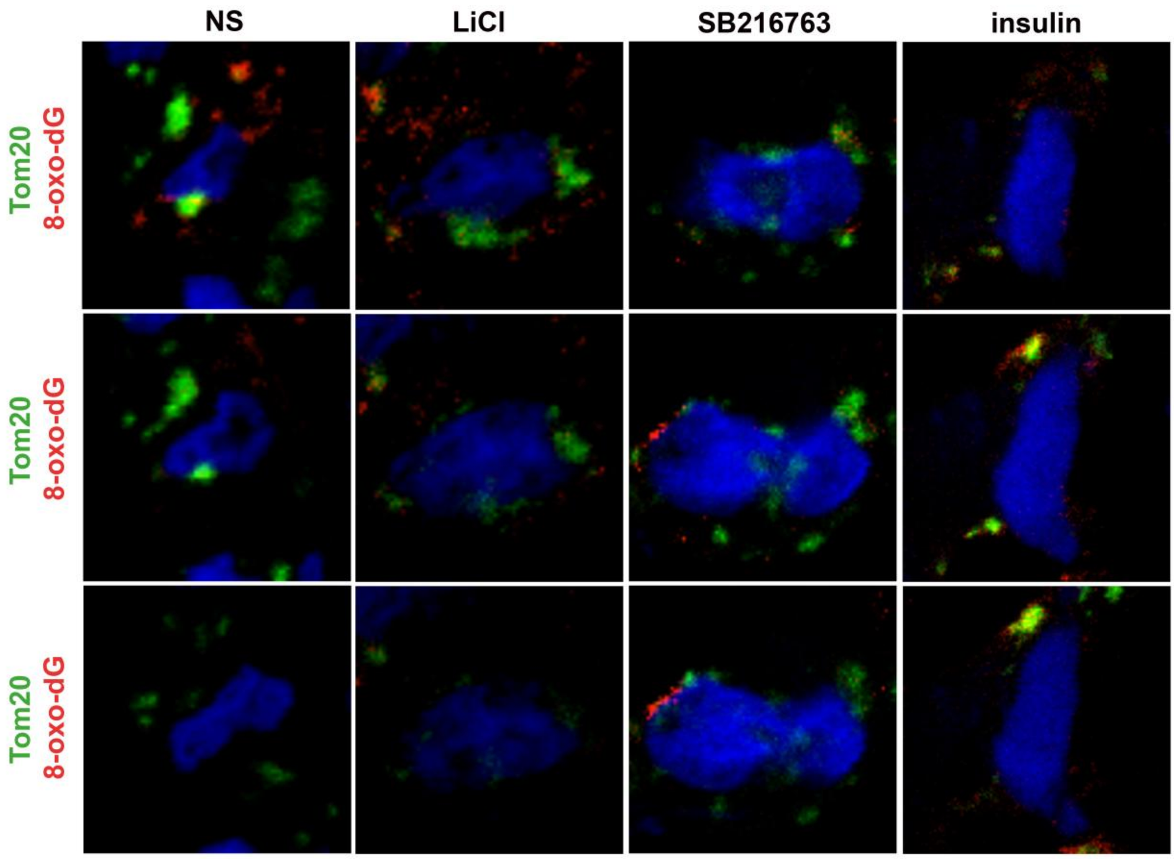

Supplementary Fig. S2. 8-oxo-dG occurs specifically in mitochondrial DNA: confocal analysis demonstrated the lack of signal in genomic DNA and 8-oxo-dG localization exclusively in the mitochondria, overlapping with the mitochondrial marker TOM20. In some cases the signal is close to the external surface of the nuclei, yet distinct from the genomic DNA and rather localized in mitochondria that are proximal to the nucleus. The figure reports details of the cells presented in the high magnification (600x) pictures in bottom rows of Figure 2 A. For each condition three distinct confocal optical sections of the nuclei are presented, at different Z-height.

S3

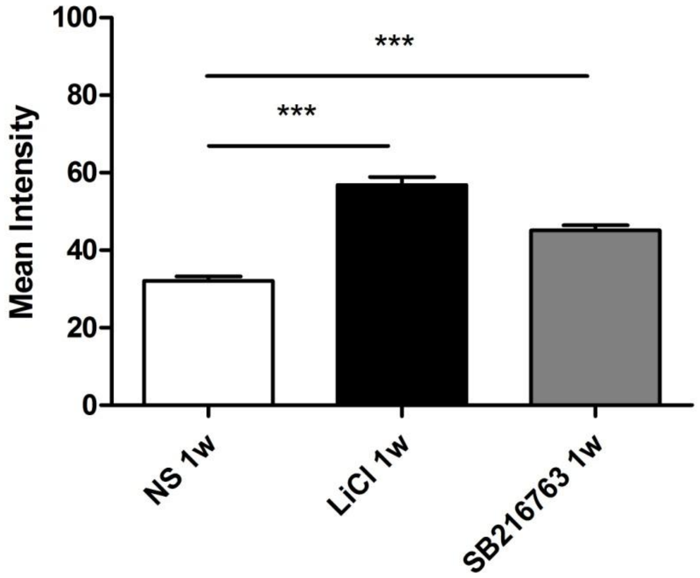

Supplementary Fig. S3. “Long term” treatment with either 5mM LiCl or 10µM SB216763 increased nuclear translocation of active β-catenin as assessed by confocal analysis of the staining intensity in the nuclear area as mapped by the nuclear probe Sybr Green. Graphs report the mean nuclear intensity of immunofluorescent staining in several 60x fields taken from sections of micromasses at 1 week of culture. The means are relative to several hundreds (n = 2137, NS; 1441, LiCl and 2043, SB216763 of different cell nuclei as evaluated by the Student’s t-test for independent samples. ***P < 0.001

S4

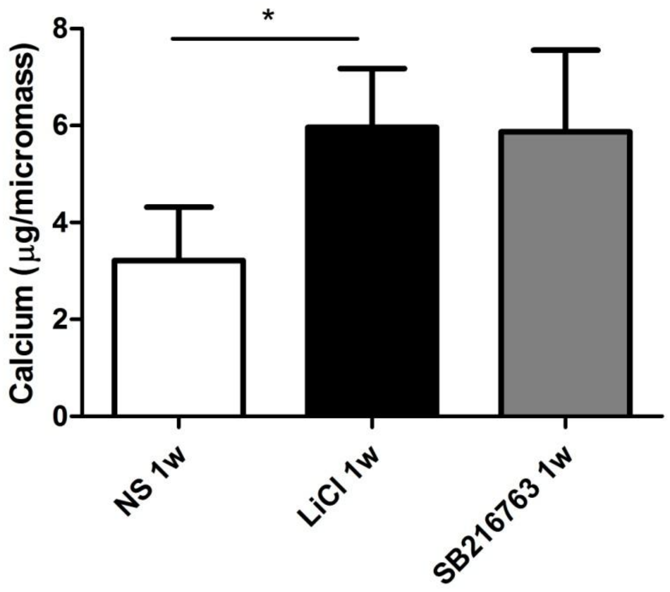

Supplementary Fig. S4. LiCl but not SB216763 treatment led to a statistically significant increased calcium deposition. The picture reports data derived from 7 experiments with 1 week micromasses established with cell of different patients (n=7, *P < 0.05). Calcium content was quantified by Quantichrom DICA-500 assay. Given the high variability of the data, the Wilcoxon signed rank test was employed to compare each GSK3β inhibiting treatment with the basal condition.

S5

A

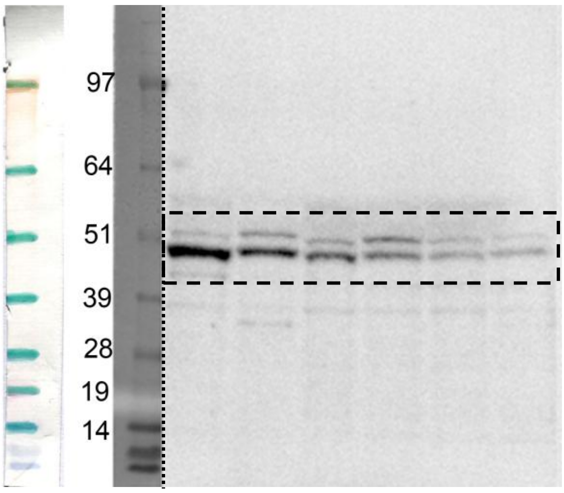
97
64
51
39
28
19
14

B

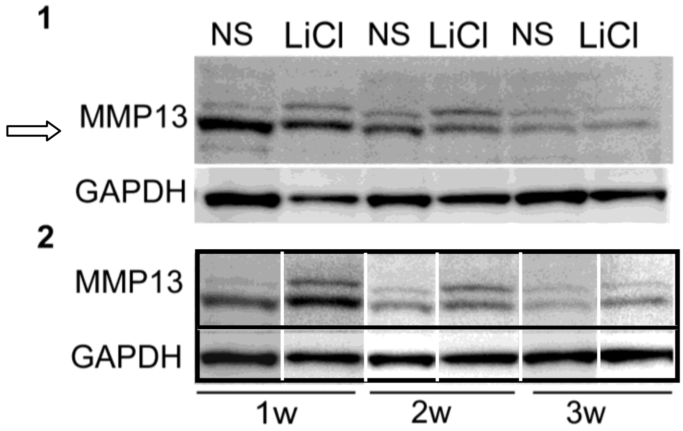
1
NS
LiCl
NS
LiCl
NS
LiCl
MMP13
GAPDH
2
MMP13
GAPDH
1w
2w
3w

C

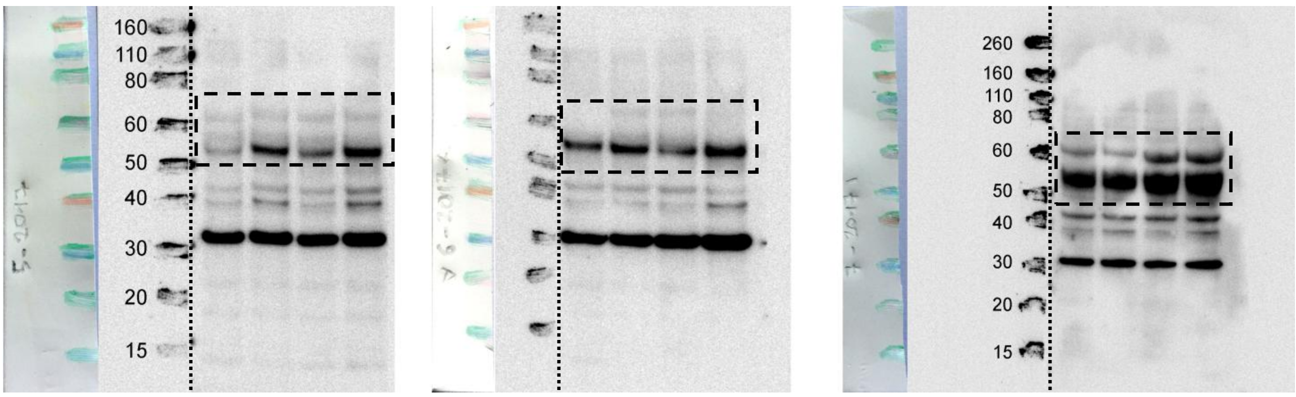
160
110
80
60
50
40
30
20
15
260
160
110
80
60
50
40
30
20
15

D

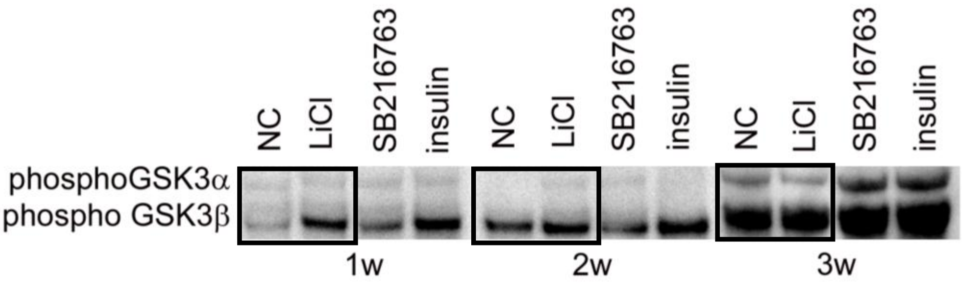
NC
LiCl
SB216763
insulin
NC
LiCl
SB216763
insulin
NC
LiCl
SB216763
insulin
phosphoGSK3α
phospho GSK3β
1w
2w
3w

E

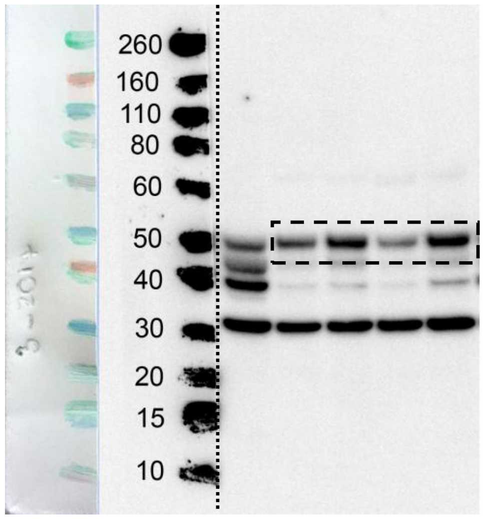

F

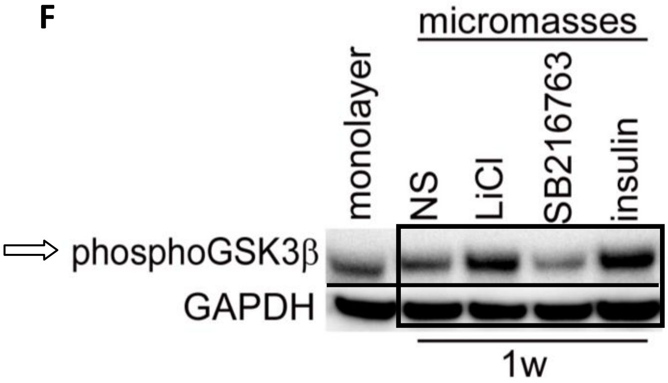

Supplementary Fig. S5. A: Full blot used to derive the MMP-13 results shown in Figure 5A of the main manuscript obtained with micromass lysates at 1-2-3 weeks in unstimulated or LiCl stimulated conditions. In this gel samples were run with NuPAGE MOPS and SeeBlue Plus2 pre-stained standards. The dashed rectangle indicates the bands included in the crop.

B: 1 Crop of the original MMP-13 results presented in full blot in A as indicated by the arrow, along with the corresponding GAPDH signal. **2**: Starting from the signals of the original acquisitions shown in 1, for each of the six samples the intensities of both MMP-13 and GAPDH signals were tuned together in order to obtain comparable intensities of the loading control across the samples, and to perform a reliable qualitative comparison of the MMP-13 signal. The white lines highlight fusion sites used to set up the modified MMP-13/GAPDH figure shown in main figure 5A and reproduced here. The black solid rectangle indicates the modified MMP-13/GAPDH results shown in Figure 5A of the main manuscript.

C: Full blots used to derive the phosphoGSK3 α and β results shown in Figure 5A of the main manuscript obtained with micromass lysates at 1-2-3 weeks in unstimulated condition or stimulated with either LiCl or SB216763 or insulin. The dashed rectangles indicate the bands included in the crops shown in D.

D: Assembly of the crops of the full blots shown in C, indicated by the dashed rectangles. The black solid rectangles indicate the bands used to setup phosphoGSK3 α and β figure in Figure 5A of the main manuscript. Therefore, in figure 5A of the main manuscript black lines highlight splicing between bands that were not adjacent in the full blot.

E: Full blot corresponding to the results of phosphoGSK3β shown in Figure 5B of the main manuscript along with its loading control and obtained with micromass lysates at 1 week in unstimulated condition or stimulated with either LiCl or SB216763 or insulin.
The dashed rectangle indicates the bands included in the crop used in figure 5B of the main manuscript. The arrow indicates the cropped bands in supplementary figure 5F.

F: Crop of the original phosphoGSK3β results shown in E as indicated by the arrow along with its corresponding GAPDH loading control. The black solid rectangle indicates the phosphoGSK3β/GAPDH results shown in Figure 5B of the main manuscript.
